# Complete Genome Sequence of the Disinfectant Susceptibility Testing Reference Strain *Staphylococcus aureus* subsp. *aureus* ATCC 6538

**DOI:** 10.1128/genomeA.00293-17

**Published:** 2017-05-11

**Authors:** Olga Makarova, Paul Johnston, Birgit Walther, Jens Rolff, Uwe Roesler

**Affiliations:** a Institute of Animal Hygiene and Environmental Health, Centre for Infection Medicine, Freie Universität Berlin, Berlin, Germany; b Evolutionary Biology, Institute of Biology, Freie Universität Berlin, Berlin, Germany; c Berlin Center for Genomics in Biodiversity Research, Berlin, Germany; d Leibniz-Institute of Freshwater Ecology and Inland Fisheries (IGB), Berlin, Germany; e Institute of Microbiology and Epizootics, Centre for Infection Medicine, Freie Universität Berlin, Berlin, Germany

## Abstract

We report here the complete genome sequence of the methicillin-sensitive *Staphylococcus aureus* subsp. *aureus* strain ATCC 6538 (FDA 209, DSM 799, WDCM 00032, and NCTC 10788).

## GENOME ANNOUNCEMENT

*Staphylococcus aureus* subsp. *aureus* strain ATCC 6538 is a standard testing strain for disinfectants (1) and was initially isolated from a human lesion prior to the introduction of the widespread use of antimicrobials (2). This strain is important not only for routine screening in antimicrobial susceptibility testing but also for studying the evolution of resistance to biocides (3). Here, we report a complete genome sequence obtained with PacBio and Illumina technologies.

The genome of strain ATCC 6538 was sequenced on a PacBio RS II sequencer using P6/C4 chemistry (10-kb insert library; average read length, 9,358 bp; 121,651 reads) and an Illumina MiSeq (TruSeq DNA PCR-free library; 300-bp paired-end reads; 545,473 read pairs). *De novo* assembly using Canu (4) produced two contigs composed of a chromosome and a plasmid, which were circularized using Circlator (5). Illumina reads were aligned to the assembly using the Burrows–Wheeler alignment method (6), and the resulting alignments were used to correct errors with Pilon (7). Gene annotation was performed using Prokka (8).

The ATCC 6538 chromosomal and plasmid genomes are 2,819,210 bp (G+C content, 32.9%) and 28,078 bp (G+C content, 30.6%) in size, respectively. The chromosomal genome possesses 2,545 open reading frames, 19 rRNAs, 62 tRNAs, and one clustered regularly interspaced short palindromic repeat (CRISPR) region. The plasmid is identical to pFDA209P (NZ_AP014943.1) and contains 30 protein-coding sequences. Two complete prophage sequences of 74.2 kb and 20.3 kb were identified using the prophage prediction server PHAST (9) and were determined to have a high degree of similarity to phages JS01 (NC_021773) and PT1028 (NC_007045.1), respectively. Sequence typing with Ridom SeqSphere+ (Ridom GmbH, Germany) (10) revealed that the strain belongs to sequence type 464, *spa*-type t3297, and is negative for *mecA*. It harbors genes encoding staphylococcal leukotoxin (LukED) and staphylococcal enterotoxins (SE)B and SEK. Overall, genetic loci associated with resistance in the phenotype were rare and included multidrug efflux pumps (e.g., *sdr*M) and *mpr*F involved in resistance to host defensin peptides.

### Accession number(s).

The complete genome sequence of *S. aureus* subsp. *aureus* strain ATCC 6538 has been deposited in GenBank under the accession numbers CP020020 and CP020021 for the chromosome and plasmid, respectively.
